# Current understanding in diagnosis and management of factor XIII deficiency

**Published:** 2013-10-22

**Authors:** M Naderi, A Dorgalaleh, Sh Tabibian, Sh Alizadeh, P Eshghi, Gh Solaimani

**Affiliations:** 1 Genetic Researcher Center in Non-Communicable Disease, Zahedan University of Medical sciences; 2 Hematology Department, Allied Medical School, Tehran University of Medical Sciences, Tehran, Iran; 3 Pediatric Congenital Hematologic Disorders Research Center, Zahedan University of Medical sciences

## Abstract

Factor XIII or "fibrin-stabilizing factor," is a transglutaminase circulates in the blood circulation as a hetero tetramer with two catalytic A subunits and two carrier B subunits. This important coagulation factor has a crucial role in clotting cascade and produces strong covalent bonds between soluble formed fibrin monomers during coagulation. This stable cross linked fibrin strands are resistanttodegradationby thefibrinolyticsystem that enablesthe bodyto stoppotential bleeding episodes. In the absence or severe decrease of factor XIII, although the clot is formed, but is rapidly degraded by the fibrinolytic system, and delayed bleedingoccurs.Factor XIII deficiency is an extremely rare bleeding disorder with estimated incidence of 1/2-3000, 000 in the general population. Presumptive diagnosis of factor XIII deficiency was by clot solubility test in 5M urea or 1% monochloroacetic acid environments. In patients with abnormal screening clot solubility test, the disease can be confirmedbymore specifictestssuch as quantitative factor XIII activity assay andFXIIIAgassay.After diagnosis of disease all patients with severe factor XIII deficiency(<1 U/dl) shouldreceive prophylactic substitution therapywith fresh frozen plasma (FFP) and cryoprecipitate as traditional choices or purified concentrateof blood coagulation factor XIII (Fibrogammin P) inorder to control severe and life-threatening clinical complications of factor XIII deficiency.

## Introduction

Coagulation factor XIII is a zymogene that at the end of coagulation cascade cross-linked unstableand loose strand of fibrin to make stable clot. It circulates in blood stream in tetrameric form consist of two catalytic subunits (A2) and two carrier subunits (B2) (1). This factor inherited in autosomal recessive manner. Factor XIII deficiency is a rare bleeding disorder leading to formation of weak, unstable clot which is easily degraded by the fibrinolytic system andrepresented on spectrum of significant clinical manifestations. Clinical features of this disease include delayed bleeding, umbilical cord bleeding, delayed cord separation, significant bleeding following circumcision and trauma, poor wound healing, recurrent spontaneous miscarriage and intracranial hemorrhage. 

Factor XIII in most of the deficient patients has no activity and also the A subunit of protein is absent in plasma, platelets and monocytes(1, 2).developing PTLD.

## History

Factor XIII first discovered in 1944 by K.C.Robbinsas a coagulation factor.Although later,several other functions for this unknown factor “serum factor”were introduced. Subsequent studies on this factor by Laki and Lorand confirmed the existence of this new factor and also its role in coagulation that lead to renaming of “serum factor. ” to fibrin stabilizing factor because in the absent of this factor, formed clot was unstable and easily degraded in weak acid or bases and 5 M urea. In 1963 at the Congress of the International Society on Thrombosis and Heamostasi, its official name as factor XIII was adopted. There have been some other names for factor XIII including Laki–Lorand or L–L factor, fibrinase, protransglutaminase and fibrin polymerase.

Initially it was suggested that factor XIII acts as a glue and sticks fibrin strands together but further studies revealed the enzymatic activity of factor XIII in coagulation cascade in manner to cross-linking of fibrin strands and make a strong fibrin clot.

Sixteen years after discovery of factor XIII, Duckert et alin 1960 described the first case with severe factor XIII deficiency in Switzerland. This case was young boy with severe bleeding diathesis and in laboratory assessments his clot was unstable in 5 M urea. This suggesting thesuggest the presence of factor XIII deficiency in this patient.

Factor XIII deficiency categorized among rare bleeding disorders and thought to be occur in about 1 in 1-3 million in general population(3,4,5).

## Structure and function

Factor XIII is a tetrameric zymogen composed of two catalytic A subunits (FXIII-A2) bound to two carrier B subunits (FXIII-B2) it is converted into an active transglutaminase (FXIIIa) by thrombin and Ca (2+) at the terminal stage of coagulation cascade(6,7).

The FXIII-A subunit (F13A) that is synthetized by megacaryocytes, monocytes and macrophages encode by a gene is composed of 15 exons and 14 introns. This gene covers a genomic region of 160 kb, and maps to 6p24–25 chromosomal region and finally encode a mature protein consisted of 731 amino acids(8). 

FXIII-A is consist of five domain including the activation peptide (residues of 1-37), β-sandwich (residues of 38-183), central domain or catalytic core region (residues 184-515), β-barrel 1 and 2(residues 516- 627 and residues 628-731, respectively). The central domain of this subunit show a catalytic triad is formed via hydrogen bond between Cys314, His373 and Asp396(9,10). The FXIII-B subunit gene (F13B) ispredominantly synthetized by hepatocytes. This part encoded by a gene located on chromosome 1q31–32.1 consist of 12 exons and 11 introns and covers a genomic region of 28 kb. Translation of this gene resulted to the mature protein consistof 641 amino acids. This subunits have a repeated domains that also named sushi-domain repeats or GP-I structures(6,11,12).

For the activation of FXIII, it converted to plasmaFXIIIabythrombin via cleavage at N-terminal activation peptide (AP-FXIII) inarginin residues. 

Then this factor miss the B subunit in the presence of Ca2+ and fibrin resulted to conformational change in this factor structureand substrate can accesses the active site cysteine(Cys311) located within the sequence Tyr-Gly-Gln-Cys-Trp. Activated FXIII-A2 then catalyses inter-molecular gamma-epsilon-lysine band between fibrin chains or other proteins with two residues of glutamine and lysine. In fact the polymerization of fibrin molecules occurs via γ-dimerization of Gln 398 in one molecule and Lys406 in another fibrin molecule. Additional Polymerization can occur through the residues of Gln328, Gln366 and Lys508 in fibrin molecules alpha chains. 

FXIII also incorporate with α2-antiplasmin and thrombin activatable fibrinolysis inhibitor(TAFI) therefore increase cross- linking of fibrin and stabilizing the clot, make it more resistant to plasmin proteolytic degradation function(6,13, 14,15).

## Clinical manifestations

Patients with congenital FXIII deficiency have an increased tendency to bleeding and most of them experience bleeding episodes from birth that continue during life. Knowledge about the clinical manifestations of FXIII deficiency is important in management of disease.

In the absent of FXIII, fibrin clots remain unstable ,breakdown easily and consequently bleeding episodes occur(16).Among these patients umbilical cord bleeding was the most common clinical presentation , deep soft tissue hematoma and prolonged wound bleeding are other common clinical manifestations. Spontaneous intracranialhemorrhage also is a common and significant clinical presentation that endangers patients' life (17). Some of these manifestations are:

**Umbilical bleeding**

Early bleeding episode occurred during the few days after birth with umbilicalbleeding that can be life threatening in homozygous individuals.Umbilical bleeding is considered as a most common clinical manifestation and can be found in approximately 80% of patients(17, 18).

**Intracranial bleeding**

Life threatening episodes such as spontaneous intracranial hemorrhage were seen in approximately 25–30% of patients while this severe feature was not seen in other rare bleeding disorders. intracranial hemorrhage is also reported as a main cause of death in this patients.(1,6,20, 21).

Intraparenchymal was the most common site of ICH and subdural hemorrhage and epidural hemorrhage were other sites of ICH (22, 23). Temporal and occipital were common anatomic regions of Intraparenchymal hemorrhage and tempro-occipital was other common region (22, 23).

**Impaired wound healing**

Delayed wound healing has been reported among 14-29% of patients first recognized with FXIII. Cross-linking of fibrin and fibronectin at the site of injury, the proangiogenic effect and the enhancement of the migration and proliferation of monocytes and fibroblasts are some contributing factors of FXIII in the process of tissue repair and wound healing.1

Impaired wound healing also occurfrequently and reported in 14-29% patients. Polymerization of fibrin molecule, proangiogenic effect and increased proliferation of cells such as monocytes and fibroblasts has an essential role in repairing tissue (1,6).

**Recurrent spontaneous miscarriages**

Recurrent spontaneous miscarriage is another bleeding complication in women with FXIII deficiency. FXIII is t required for attachment of the cytotrophoblasts after invading to the endometrium. Thus in FXIII deficiency the likelihood of miscarriage increases and consequently bleedings occur (20,21).

**Subcutaneous and soft tissue bleeding**

Subcutaneous bleeding due to trauma or bruises isanother bleedingepisode thatoccurs in FXIII deficient patients. Mouth and gums bleeding is also occur frequently and were seen in approximately 30% of individuals. In addition Muscles and joints bleeding reportedin many cases( 1,6,19).

**Other bleeding episodes**

There are another frequent features that were seen in FXIII deficient patients including mucosal tract bleeding(particularly epistaxis and menorrhagia), Echymoses and postoperative hemorrhages(1,6,22).

**Diagnosis:**

**Laboratory assay**

FXIII deficiency can be initially diagnosed by observing bleeding episode with normal routine clotting tests including prothrombin time (PT), activated partial thromboplastintime (aPTT), fibrinogen level, platelets count and bleeding time (BT) (13).

**Qualitative assay**

Diagnosis of the disease based on solubility of blood clot in solution of 5 M urea or 2% acetic acid (or1% monochloracetic acid). These tests are qualitative tests andshow positive result if the activity of FXIII in plasma of the patients is absent or close to zero. If the result of test become positive subsequently quantitative analysis of FXIII activity is needed (23, 24).

**Quantitative assay**

In the condition that the result of urea clot solubility test becomes positive, a quantitative analysis of FXIII activity must be performed. There are different quantitative assay as follow:

**Photometric methods**

For this mean 2 kits are available including the Berichrom FXIII (Dade Behring, Marburg, Germany) and the REA-chrom FXIII (Reanal, Budapest, Hungary. ). These two kits work based on ammonia releasein the transglutaminase reaction.

**Amine incorporation assay**

This method performed by using Pefakit Factor XIII kit (Pentapharm, Basel, Switzerland) and based on measuring the amines covalently cross-linked to a protein substrate. This test is sensitive to theVal34Leu polymorphism and show a high level of activity of FXIII in this condition.

**Fluorometric assay**

The measurementof FXIII level by this method performed by N-zymeBioTec kit (Darmstadt, Germany) based on isopeptidase activity of FXIIIa.

**Antigenic ELISA techniques**

Inthis method concentrations of the A and B subunits is evaluated. Normal range of plasma FXIII activity is 53.2%-221.3% (mean 105% ± 28.56% SD) (25,26)

**Prenatal diagnosis**

Due to inadequate management in patients who suffer from rare bleeding disorders such as FXIII deficiency, they rarely live beyond childhood. In order to prevent the birth of suffered children, molecular characterization, carrier detection and prenatal diagnosis are the key solutions.

## Treatment

**The best scheme of treatment**

The treatment of bleeding episode on this deficiency is based on half life of FXIII (11-14 days) therefore replacement material should be used at large interval (20-30 days). Replacement therapy in this deficiency can be administered through fresh frozen plasma ( preferably virus-inactivated) in doses of 10 mL kg−1in 4–6 weeks interval , cryoprecipitate (cryo) administered in doses of 1 bag per 10–20 kg every 3–4 weeks and pasteurized FXIII concentrates(about 240 units/vial). Among theseitems,the virus-inactivated FFP and particulary pasteurized concentrates are preferred. The first FXIII from human source that used in replacement therapy was produced from placenta (Fibrogammin HS®) but later this product replaced by plasma extracted FXIII concentrates [Fibrogammin P® (CSL Behring, Marburg, Germany) and FXIII-BLP® (Bio-Product Laboratory, Elstree, United Kingdom)]. 

In addition recombinant FXIII now are available(Novo Nordisk, Bagsvaerd, Denmark).(6,29,30, 31).

**Complications of treatment**

Among rare bleeding disorder (RBDs) patients with FXIII and FV deficiency may be develop alloantibodies due to treatment with FFP and FXIII concentrates.

In addition, in some cases the occurrence of inhibitors are also reported in a European questionnaire survey (31, 33).

**Treatment in women: Pregnancy, Delivery**

Management of pregnantwomen with FXIII deficiency requires close monitoring due to the high risk of abortion and pregnancy loss. Therefore prophylactic therapy before 5–6 weeks of gestation is recommended. The minimum FXIII plasma level of 10% is essential for reducing the risk of pregnancy loss. To maintain this level of FXIII, administration of 250 IU/week of FXIII until the 22nd weeks of gestation and increasing the dosage to 500 IU/week from the 23rd weeks of gestation is recommended. FXIII levels should be maintained greater than 30% by use of a dosage of (1000 IU) at the time of delivery (20,32,31).

**Treatment in surgery**

In surgical procedures, in order to manage bleeding episodes, FXIII should be keptin levels greater than 10–20%. 

In major and minor surgery administration of 20– 30 U/kg per day andthen 10–20 U/kg ofFXIII per day for 2-3 days is recommended. The goal of therapy in major surgery is achieving the level of FXIII over 5% to promote wound healing (6,35,30).

**Acquired FXIII deficiency**

Acquired FXIII deficiency is a rare bleeding disorder thatoccur due to shortened production, increased consumptionare autoantibodies mediated destruction of FXIII subunits. It is described in various diseases including some type of leukemias, liver disease, Crohn’s disease, disseminated intravascular coagulation(DIC), different form of colitis, Henoch- Schonleinpurpura ,inflammatory bowel disease, , systemic lupus erythematosus, pulmonary embolism, erosive gastritis ,sepsis, stroke and major surgeries.These patients rarely need replacement therapy (6,13, 14,36).

**Molecular genetics**

Factor XIII deficiency accompanied by mutations in the genes of factor XIII A or B subunits. The mutations in A subunit are more studied based on catalytic function of factor XIII-A subunit and also associated with significant clinical manifestations. First mutation in factor XIII was reported in 1992. 

According to available data on Cardiff database (http://www.hgmd.cf.ac.uk), Factor XIII Registry Database website (http://www.f13-database.de ) and other databases more than mutations were identified in factor XIII gene.

Missense mutations are the most common in both subunits and accounting for ~50% of all factor XIII mutations. Other mutations such as nonsense mutations and frame - shift mutations in this subunitalso have been reported.

Most of the mutations occur in catalytic A subunit which is attributed to its large size and important role in activity of enzyme. The interaction site of two A subunits which is necessary for dimerization is another site with a few number of mutations caused alteration in transglutaminase activity of Factor XIII. A minority of mutations are in carrier B subunit.

Most mutations in gene of factor XIII A lead to abnormal folding and instability of produced protein. (1,7,37).

The most common factor XIII-A polymorphisms are Val34Leu in exon 2, Tyr204Phe in exon 5, Pro(CCA)331(CCC)Pro in exon 8, Pro564Leu ,Glu(GAA)567Glu(GAG) in exon 12, Val650Ile , Glu651Gln in exon 14. In factor XIII-B subunit two common polymorphisms were reported, His95Arg and C29759G change in intron K29756 which later lead to a novel splice site receptor(36,37).

**Val34leu: **Val34Leu is one of the most common polymorphism of factor XIII which was first described by Mikkola et al. This polymorphism is located 3 residues prior to the thrombin cleavage site and has been shown to accelerate the activation rate by 2.5 times in its presence. This polymorphism has been widely studied and also their associations with many diseases such as risk of thrombosis, Myocardial infarction (MI), coronary artery disease (CAD) have been evaluated. The first case-control study on the association of factor XIII-A val34leu polymorphism and risk of CAD and MI was done by Kohler et al in 1998. They revealed a protective effect of this polymorphism aginst risk of MI(7,38,39).

**Trp187Arg: **Trp187 amino acid locatedin FXIIIA sequence is conserved in all known transglutaminase. The wild type of Trp187 is located towards the surface of the protein between the catalyticcore and the b-sandwich domain. Substitution of Trp187 with arginine in factor XIII-A subunit resulted to decreased catalytic activity and also instability of factor XIII. The TGG→CGG mutation which lead to Trp187Arg change is one of the most common mutations in Iranian patients (40).

Trp187Arg is the most common mutation of FXIII-A subunite in southeast of Iran and probably due to the large numberofpatients in thisarea (352 patients), is the most common mutation of factor XIII worldwide (22).

**Tyr204Phe: **This common polymorphism of factor XIII-A lead to decreasesof FXIII plasma level and activity. There is a strong association between Tyr204Phe polymorphism and ischemic stroke that enhance the risk of disease by nearly 9-fold (7).

**Pro564Leu:**This is another common polymorphism among patients with factor XIII deficiency and similar manner to Tyr204Phe polymorphism lead to decreases of FXIII plasma level and activity .unlike Tyr204Phe mutation it does not increases the risk of ischemic strokes(7).

**Arg77His: **The wild type Arg residue is critical since in the crystal structure it is observed to be part of an 

extended H-bond network involves His64, His65 and Phe184. Substitution of Arg77with His would disrupt this network since they will not be able to fill in for the large Arg side chain. This substitution lead to highly unstability of factor XIII protein (41).

**Table I:** A number of factor XIII mutations

	**Nucleotide Exchange**	**Codon, amino acid change**	**Exon **	**Affected Domain**	**Mutation type**
**South East Iran**	c.559T>C	Trp187Arg	4	Core	Missense
**South Indian(Asian)**	c.210T>G	Tyr69X	3	Beta sandwich	Nonsense
**South Indian(Asian)**	c.791C>T	Ser263Phe	6	Core	Missense
**South Indian(Asian)**	c.892_895dupG	Ser290/Ala291fs	7	Core	Duplication
**Algerian-French**	ins T866	Pro255	6	Core	Insertion
**Dutch 3 **	(-7 to -20)InsTT	-	2	Activation Peptide	Insertion
**Dutch 2 **	c.949G>T	Val316Phe	7	Core	Missense
**Nagoya I**	CCTGCAGGTAAGCCACCGCCdel	-	1/Intron 1	Activation Peptide	Deletion
**Swedish C-II-3**	c.27delT	-	2	Activation Peptide	Deletion
**Danish D-I-2**	c.27delT	-	2	Activation Peptide	Deletion
**Finnish FII **	IVS3+6 T>C	-	Intron 3	Beta sandwich	Splice site substitution
**Finnish EII**	c.728T>C	Met242Thr	6	Core	Missense
**German A-III-4 **	c.758G>T	Arg252Ile	6	Core	Missense
**German A-II-10**	c.980G>A	Arg326Gln	8	Core	Missense
**German A-III-4**	c.980G>A	Arg326Gln	8	Core	Missense
**Swedish B-II**	c.1496T>C	Leu498Pro	12	Core	Missense
**Finnish AII**	c.1984C>T	Arg661X	14	Barrel-2	Nonsense
**Finnish BII**	c.1984C>T	Arg661X	14	Barrel-2	Nonsense
**Finnish CII**	c.1984C>T	Arg661X	14	Barrel-2	Nonsense
**Finnish DII**	c.1984C>T	Arg661X	14	Barrel-2	Nonsense
**Finnish FII**	c.1984C>T	Arg661X	14	Barrel-2	Nonsense
**Finnish EII **	c.1984C>T	Arg661X	14	Barrel-2	Nonsense
**Swedish B-II **	c.1984C>T	Arg661X	14	Barrel-2	Nonsense
**Japanese**	c.131-132delAG	-	Intron 2/3	Activation peptide	Splice site deletion
**Family S.**	c.183C>A	Asn60Lys	3	Beta sandwich	Missense
**Family J.**	c.1504G>A	Gly501Arg	12	Core	Missense
**Family S. **	c.1326C>A	Tyr441X	11	Core	Nonsense
**Yorkshire Family 03**	c.183C>A	Asn60Lys	3	Beta sandwich	Missense
**Yorkshire Family 04 **	c.291-292delGG, c.291-296insTCGTCC	-	3	Beta sandwich	Deletion/Insertion
**D6 (UK)**	c.319G>T	Gly106	3	Beta sandwich	Splice site substitution
**Yorkshire Family 01**	c.319G>T	Gly106	3	Beta sandwich	Splice site substitution
**Yorkshire Family 02 **	Gross Deletion (>100kb)	-	3 and 12	Core	Deletion
U.K.*	c.1626C>G	Asn541Lys	12	Barrel-1	Missense
**Yorkshire Family 05**	c.2045G>A	Arg681	14	Barrel-2	Splice site substitution
**C5 (UK)**	c.2075G>A	Trp691X	15	Barrel-2	Nonsense
**U.K.**	IVS5-1 G>A	-	Intron 5	Core	Splice site substitution
U.K.*	IVS5-1 G>A	-	Intron 5	Core	Splice site substitution
**C5 (UK)**	c.709delG	-	6	Core	Deletion
**North Pakistan**	c.888C>G	Ser295Arg	7	Core	Missense
**B3, B4 (Pakistan)**	c.888C>G	Ser295Arg	Intron 7	Core	Missense
U.K.**	IVS7+1 G>A	-	Intron 7	Core	Splice site substitution
**A1, A2 (Pakistan)**	c.979C>T	Arg326X	8	Core	Nonsense
**Pakistan**	c.1064T>C	Leu354Pro	8	Core	Missense
**Yorkshire Family 02**	c.1226G>A	Arg408Gln	10		Missense
**Chinese 2**	c.232C>T	Arg77Cys	3	Beta sandwich	Missense
**Chinese 3**	c.599-600delAA	-	5	Core	Deletion
**Chinese 1**	c.1241C>G	Ser413Trp	10	Core	Missense
**Swiss 3**	c.232C>T	Arg77Cys	3	Beta sandwich	Missense
**Swiss 4**	c.232C>T	Arg77Cys	3	Beta sandwich	Missense
**Swiss 5**	c.232C>T	Arg77Cys	3	Beta sandwich	Missense
**Swiss 12**	c.232C>T	Arg77Cys	3	Beta sandwich	Missense
**Swiss 14**	c.232C>T	Arg77Cys	3	Beta sandwich	Missense
**Swiss 6**	c.479T>G	Met159Arg	4	Beta sandwich	Missense
**Swiss 10**	c.479T>G	Met159Arg	4	Beta sandwich	Missense
**Swiss (Serbian) 13**	c.646G>A	Gly215Arg	5	Core	Missense
**Swiss 2**	c.781C>T	Arg260Cys	6	Core	Missense
**Iranian B**	c.233G>A	Arg77His	3	Beta sandwich	Missense
**Iranian C**	c.233G>A	Arg77His	3	Beta sandwich	Missense
**Iranian G **	c.233G>A	Arg77His	3	Beta sandwich	Missense
**Iranian H **	c.233G>A	Arg77His	3	Beta sandwich	Missense
**Iranian L**	c.233G>A	Arg77His	3	Beta sandwich	Missense
**Iranian D**	c.689delA	-	5	Core	Deletion
**Iranian A**	c.781C>T	Arg260Cys	6	Core	Missense
**Iranian I **	c.782G>A	Arg260His	6	Core	Missense
**Iranian F **	c.1149G>T	Arg382Ser	9	Core	Missense
**Iranian E**	c.2066 del C	-	14	Barrel-2	Deletion
**Greek**	c.249-261delCTATGTGCAGATT	-	3	Beta sandwich	Deletion
**South Pakistan 6**	c.980G>A	Arg326Gln	8	Core	Missense
**Greek (Crete)**	c.1201insC	-	9	Core	Insertion
**Malaysian Indian**	c.1243G>T	Val414Phe	10	Core	Missense
**Canadian English (Calgary)**	c.2003T>C	Leu667Pro	14	Barrel-2	Missense
**South Pakistan 1**	c.2045G>A	Arg681	14	Barrel-2	Splice site substitution
**South Pakistan 2**	c.2045G>A	Arg681	14	Barrel-2	Splice site substitution
**South Pakistan 3**	c.2045G>A	Arg681	14	Barrel-2	Splice site substitution
**South Pakistan 4**	c.2045G>A	Arg681	14	Barrel-2	Splice site substitution
**South Pakistan 5**	c.2045G>A	Arg681	14	Barrel-2	Splice site substitution
Japanese*	c.397insG	-	4	Beta sandwich	Insertion
**Bristol 1**	c.514C>T	Arg171X	4	Beta sandwich	Nonsense
**Family B**	c.1064T>C	Leu354Pro	8	Core	Missense
**Family A**	c.1196C>A	Thr398Asn	9	Core	Missense

**Figure 1:**
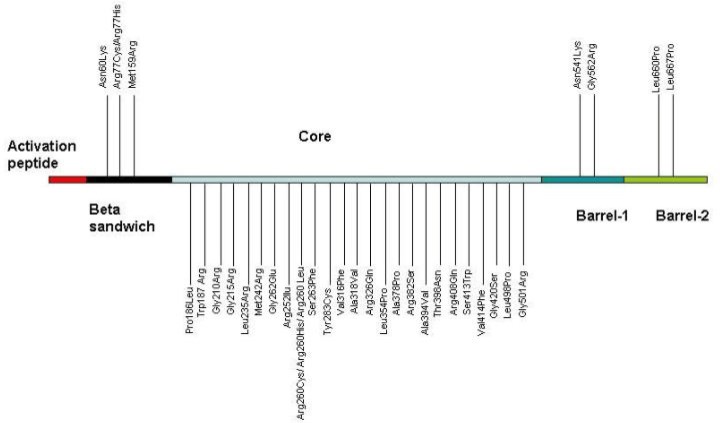
The most frequent mutations of factor XIII (According to http://www.f13- database.de).
